# The carotenoid redshift: Physical basis and implications for visual signaling

**DOI:** 10.1002/ece3.10408

**Published:** 2023-09-07

**Authors:** Dakota E. McCoy, Allison J. Shultz, Jacqueline E. Dall, Jennifer A. Dionne, Sönke Johnsen

**Affiliations:** ^1^ Department of Materials Science and Engineering Stanford University Stanford California USA; ^2^ Hopkins Marine Station Stanford University Pacific Grove California USA; ^3^ Department of Biology Duke University Durham North Carolina USA; ^4^ Ornithology Department Natural History Museum of Los Angeles County Los Angeles California USA; ^5^ Department of Radiology Stanford University Stanford California USA

**Keywords:** honest signal, hue, light absorbance, optics, pigment, sexual selection

## Abstract

Carotenoid pigments are the basis for much red, orange, and yellow coloration in nature and central to visual signaling. However, as pigment concentration increases, carotenoid signals not only darken and become more saturated but they also redshift; for example, orange pigments can look red at higher concentration. This occurs because light experiences exponential attenuation, and carotenoid‐based signals have spectrally asymmetric reflectance in the visible range. Adding pigment disproportionately affects the high‐absorbance regions of the reflectance spectra, which redshifts the perceived hue. This carotenoid redshift is substantial and perceivable by animal observers. In addition, beyond pigment concentration, anything that increases the path length of light through pigment causes this redshift (including optical nano‐ and microstructures). For example, male *Ramphocelus* tanagers appear redder than females, despite the same population and concentration of carotenoids, due to microstructures that enhance light–pigment interaction. This mechanism of carotenoid redshift has sensory and evolutionary consequences for honest signaling in that structures that redshift carotenoid ornaments may decrease signal honesty. More generally, nearly all colorful signals vary in hue, saturation, and brightness as light–pigment interactions change, due to spectrally asymmetrical reflectance within the visible range of the relevant species. Therefore, the three attributes of color need to be considered together in studies of honest visual signaling.

## Graphical abstract

Carotenoid pigments appear redder as the concentration of a pigment increases. The carotenoid redshift arises due to the asymmetrical reflectance spectra of carotenoid‐colored ornaments and the exponential attenuation of light. Structures that increase the path length of light through carotenoid pigment also cause a redshift, as we demonstrate in the colorful *Ramphocelus* tanagers.

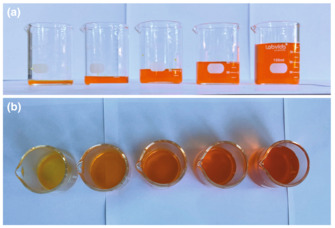

## 1 INTRODUCTION

**FIGURE 1:**
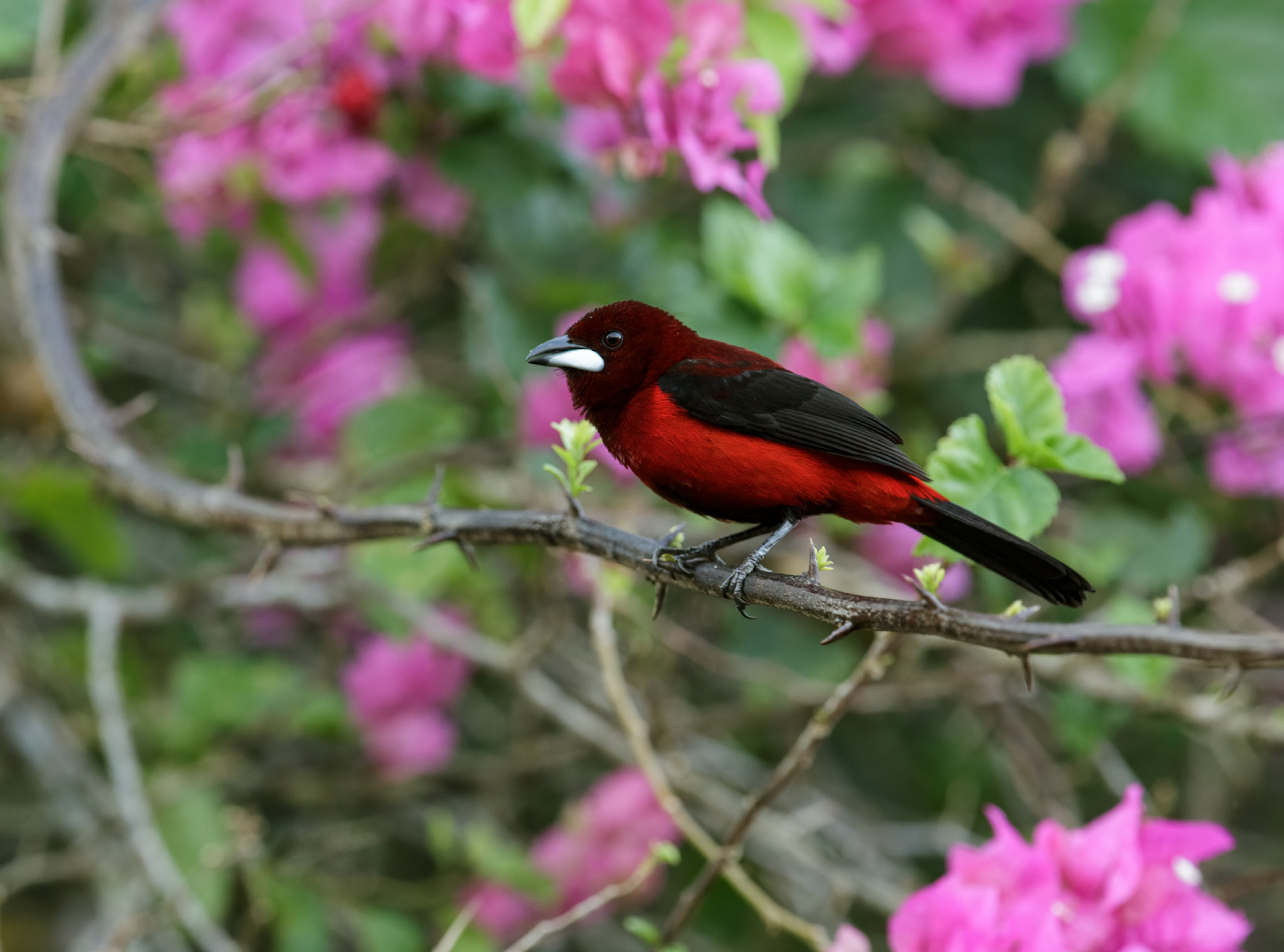
A male Crimson‐backed Tanager, *Ramphocelus dimidiatus*, flaunts red feathers colored by carotenoid pigments. This bird was photographed in Cerro Azul, Panama province, Panama by Nick Athanas, antpitta.com.

Carotenoids are an important class of pigments for animal coloration (Hill, 1991), plant biology (Nisar et al., 2015), and photosynthesis (Frank & Cogdell, 1996). Many colorful animals owe their striking red, orange, and yellow ornaments to carotenoid pigments (Figure 1), and researchers often consider carotenoids to be a classic example of honest signaling (Weaver et al., 2018). Honest signaling is a theory of sexual selection by which colorful signals convey an individual's quality to potential mates (Endler, 1978; Folstad & Karter, 1992; Koch et al., 2019; Simons, Cohen, et al., 2012; Zahavi, 1975). Briefly, carotenoids in vertebrates: (1) must be eaten rather than synthesized; (2) are correlated with many (but not all) individual quality measures (Koch et al., 2018; Simons, Cohen, et al., 2012; Weaver et al., 2018); and (3) are interpreted either as a costly signal to produce or as an index of proper metabolic function (Cantarero & Alonso‐Alvarez, 2017; Hill & Montgomerie, 1994; Weaver et al., 2017). Honest signaling theory is one among many mutually compatible hypotheses about sexual selection (e.g., arbitrary esthetic evolution (Prum, 2012), runaway selection (Fisher, 1999), sensory bias (Dawkins & Guilford, 1996), or species identification (Hill, 2015)). However, honest signaling has been invoked as a primary explanation for colorful ornaments, particularly when mediated by carotenoids (Hill, 2022).

Researchers often test honest signaling theory through case studies on carotenoid pigmentation in birds (e.g., Cantarero & Alonso‐Alvarez, 2017; Hill et al., 2019; Hill & Montgomerie, 1994; Weaver et al., 2017) and poecilid fish (e.g., Candolin, 1999; Grether et al., 1999, 2001; Pike et al., 2007; Wedekind et al., 1998). Most often, researchers measure color as a proxy for carotenoid content, with the hypothesis that regions with more saturated or redder values are signals of a higher quality individual (see discussion by Saks et al., 2003; Weaver et al., 2018). Therefore, it is important to understand the physical basis of color/hue, saturation, and brightness in carotenoid‐colored ornaments.

Carotenoid‐colored signals tend to appear redder as pigment concentration increases, as many researchers have noted (Brush, 1970; Butler et al., 2011; Saks et al., 2003; Troy & Brush, 1983). However, pigment concentration is not the only driver of this carotenoid redshift: structural changes to tissue can increase the path length of light through pigment and therefore make ornaments appear redder (see Section 2.3). In one study, researchers found that carotenoid pigment content only explains about 32%–51% of variation in chroma and hue (Saks et al., 2003). Therefore, to make proper conclusions about sexual selection, we need a more complete understanding of the relationship between carotenoid pigment concentration, tissue structures, and coloration.

Here, we analyze the carotenoid redshift using both theory and data. First, we explain why carotenoid‐colored ornaments redshift with higher pigment concentration. Second, we show that the redshift is perceptually significant, not only for humans but also for birds based on visual modeling. Third, we demonstrate that pigment concentration is not the only driver of the carotenoid redshift: carotenoid‐colored ornaments redshift when structural modifications increase the path length of light through pigmented tissue, as illustrated by an example from tanager birds. Fourth, we discuss the implications of these findings for mate choice and honest signaling.

## 2 METHODS

To explain the physical basis of the carotenoid redshift (Section 2.1), we draw upon optical theory using simplified and idealized equations for absorbance and reflectance (Box 1, Johnsen, 2012).

To determine whether the carotenoid redshift is substantial and perceivable (Section 2.2), we simulate changes in reflectance for carotenoid‐bearing tissues by manipulating real reflectance spectra of a chlorophyll‐colored leaf and two carotenoid‐colored birds (Data S1). Specifically, we raise the reflectance data to the power of the simulated change in concentration (e.g., *R* ^2^ simulates doubling the concentration). It is important to note that this method considers the entire system as a whole, consisting of carotenoids within tissue. With these original and modified spectra that represent simulated concentrations ranging from 0.25×–2×, we calculate two measures of perceived hue. We calculated the quantum catches for each color cone in an average avian UV‐visual system using *vismodel* in the R package *pavo* with an ideal illuminant equal to 1 at all wavelengths (Maia et al., 2019). Results did not differ substantially when we repeated this analysis (1) for a non‐UV sensitive avian visual system and (2) across three additional illumination conditions, shaded forest shade, blue sky, and daylight (Data S1).

**FIGURE 2:**
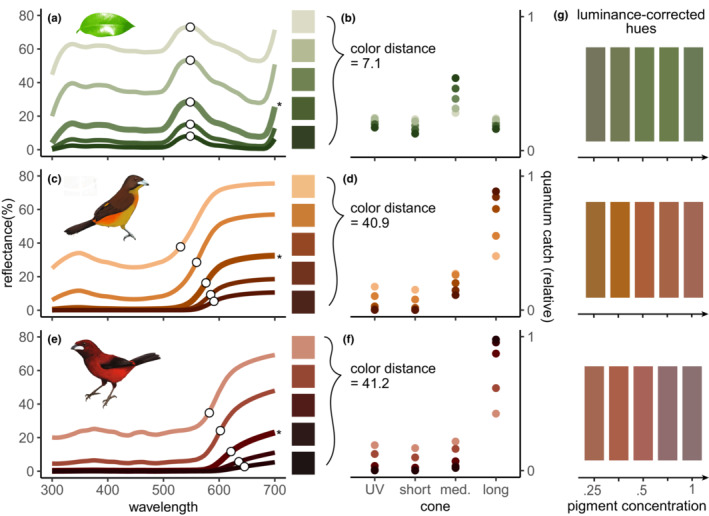
Carotenoid signals appear redder with higher pigment concentration (not just darker and more saturated) due to their asymmetrical reflectance spectra. Here we plot reflectance spectra of (a) a green leaf and (c, e) two carotenoid‐pigmented birds as well as simulated relative quantum catch per cone of a tetrachromatic bird perceiving those spectra (b, d, f). The thickest line (marked with an asterisk on the right) in each panel is the measured reflectance, while the thinner lines simulate changes in pigment concentration from the least (top line in each panel) to the greatest (bottom line in each panel). (a) A green chlorophyll‐colored leaf does not appreciably change hue as pigment concentration increases. A correlate of hue in a Gaussian‐shaped spectrum, the wavelength at maximum reflectance, stays at 548 nm. (b) To a tetrachromatic bird, visual modeling shows that the leaf changes hue by 7.1 JNDs (just noticeable differences) as the concentration of chlorophyll increases from 0.25× to 2×. (c) The carotenoid‐colored orange rump of a female *R. flammigerus* tanager bird changes hue to become redder at greater simulated pigment concentration. The wavelength at half‐maximum reflectance, a correlate of perceived hue in spectra of this sort, increases from 531 to 591 nm—a change from orange to orange‐red. (d) To a UV‐sensitive tetrachromatic bird, the rump appears significantly redder as pigment concentration increases from 0.25× to 2× (color distance = 41 JNDs). (e) The carotenoid‐colored red rump of a male *R. dimidiatus* tanager bird changes hue to become redder at greater simulated pigment concentrations. In this case, the wavelength at half‐maximum reflectance increases from 582 to 646 nm—a change from orange‐red to deep red. (f) To a UV‐sensitive tetrachromatic bird, the rump appears significantly redder as pigment concentration increases from 0.25× to 2× (color distance = 41.2 JNDs). (g) To better visualize the change in hue, here we reproduce luminance‐corrected color swatches of the simulated spectra in a, c, and e. Results in a, c, and e were consistent when we varied the avian visual system and illuminant (see Data S1). Spectra in (a) are from Johnsen et al. (2006) (Table S1); spectra in (b, c) are from McCoy et al. (2021) (Table S2); leaf silhouette in (a) is from yves_guillou (openclipart.org; public domain); bird silhouette artwork in (b) was created by Gabriel Ugueto and modified by Allison Shultz; bird silhouette artwork in (c) was created by Gabriel Ugueto. The five lines in each panel represent relative concentrations of 0.25, 0.5, 1, 1.5, and 2, where “1” is that of the measured spectrum.

We calculated color distance based on a receptor noise–limited model (Vorobyev & Osorio, 1998) using *coldist*. We plotted the quantum catch in Figure 2 with relative values, which sum to 1, but we used the actual quantum catch values to calculate the color distance. Points and lines in Figure 2 were colored according to a model of what color humans would see these objects to be (using *spec2rgb* in *pavo*; Maia et al., 2019).

To assess the magnitude of a carotenoid redshift in an experimental model system of sexual selection, we reanalyzed data from McCoy et al. (2021) on *Ramphocelus* tanagers. The original data are reflectance data from museum specimens selected from the Ornithology Collection at the Harvard Museum of Comparative Zoology. We analyzed data from 20 individuals, one male and one female from each of 10 species: crimson‐collared Tanager, *Ramphocelus sanguinolentus*; masked crimson tanager, *Ramphocelus nigrogularis*; crimson‐backed tanager, *Ramphocelus dimidiatus*; Huallaga tanager, *Ramphocelus melanogaster*; silver‐beaked tanager, *Ramphocelus carbo*; Brazilian tanager, *Ramphocelus bresilius*; Passerini's Tanager, *Ramphocelus passerinii*; Cherrie's tanager, *Ramphocelus costaricensis*; flame‐rumped tanager, *Ramphocelus flammigerus*; lemon‐rumped tanager, *Ramphocelus flammigerus icteronotus*. The original data were collected using an OceanOptics PX2 with a pulsed xenon light source, OceanOptics USB4000, and an OceanOptics Spectralon White Standard. The integration time was 100 ms, five scans were averaged, and the boxcar width was set to five.

In our analysis and plotting, we used R libraries *dplyr* (Wickham et al., 2023), *tidyr* (Wickham, 2017), *ggplot2* (Wickham, 2016a), *stringr* (Wickham, 2019), *beepr* (Bååth & Dobbyn, 2018), *phytools* (Revell, 2012, 2013), *pavo* (Maia et al., 2019), and *scales* (Wickham, 2016b).

All data are presented as supplemental files: Table S1 (McCoy et al., 2021) and leaf reflectance is in Table S2 (Johnsen et al., 2006). All code is available in Data S1.

## 3 DISCUSSION

### 3.1 Carotenoid‐based signals redshift as pigment concentration increases due to their asymmetrical reflectance spectra

Carotenoid‐colored signals redshift as pigment concentration increases. As birds deposit more of the same carotenoid pigments in their feathers, the feathers appear not only darker and more saturated but also redder in color. Ornithologists and color‐science researchers have observed the carotenoid redshift (Brush, 1970; Butler et al., 2011; Saks et al., 2003; Troy & Brush, 1983), but little has been written on its physical basis. In short, the carotenoid redshift arises because with more pigment, an ornament's asymmetrical reflectance spectrum changes in shape as light is exponentially absorbed (Box 1, Figure 2c–e).

BOX 1 **Light attenuates exponentially, so pigmented objects change color with pigment concentration.**

The fraction of light absorbed $f_{A}$ in a pigmented material can be expressed as:

$$f_{A}=1-e^{-\mathrm{ad}}$$

where *a* is the absorbance of the material and *d* is the optical path length through the material. In homogeneous substances, *d* is the thickness of the material along the path of the light, and *a* is linearly proportional to the concentration of the pigment (Johnsen, 2012). Thus, a higher concentration of pigment has the same effect as a longer path length. Here, we use the base of the natural logarithm, *e*, but it is also appropriate and common to use base 10 measurements of absorbance, which are called optical densities (e.g., Goldsmith & Butler, 2005; Lind et al., 2017).

For the leaf and bird feathers of interest here (Figure 2), we assume that the leaf and the layers of feathers on the bird are thick enough to not transmit a significant amount of light, and thus all light is either absorbed or reflected. Therefore, the fraction of light that is reflected back to our eye *f*_R_ (“reflectance” in Figure 2) is approximately:

$$f_{R}=1-f_{A}=1-\left(1-e^{-\mathrm{ad}}\right)=e^{-\mathrm{ad}}$$

Thus, the reflectance at each wavelength depends exponentially on *a* and *d*, our parameters representing pigment concentration and path length, respectively.

Low‐reflectance regions of a spectra (i.e., those that have a high absorbance) are more strongly affected by pigment concentration. Consider a red bird feather that reflects *f*_R_red_ = 60% of red light and *f*_R_blue_ = 20% of blue light for a given pigment concentration. If you doubled the concentration of the pigment, the fraction of red light reflected will drop from 60% to 36%.

$$f_{R\_\mathrm{red}}=e^{-\mathrm{ad}}=0.6$$

$$f_{R\_\mathrm{red}\_\mathrm{new}}=e^{-2\mathrm{ad}}={\left(e^{-\mathrm{ad}}\right)}^{2}=0.6^{2}=0.36$$

But blue reflectance will drop from 20% to 4%.

$$f_{R\_\text{blue}}=e^{-\mathrm{ad}}=0.2$$

$$f_{R\_\text{blue}\_\mathrm{new}}=e^{-a\left(2d\right)}={\left(e^{-\mathrm{ad}}\right)}^{2}=0.2^{2}=0.04$$

Because of this, the shape of the reflectance curve will change such that lower‐reflectance regions are disproportionately affected as shown in Figure 2. Specifically, the slope of the reflectance curve from ~500 to 700 nm changes substantially (and, therefore, the relative quantum catch per cone; see Figures 1 and 2).

We note that as absorbance $f_{A}$ approaches 1 (and reflectance $f_{R}$ approaches 0), any change in pigment concentration may have a negligible effect on color perception.

Carotenoid‐based signals have highly asymmetrical reflectance spectra (Johnsen, 2012) compared to those mediated by certain other common natural pigments (e.g., chlorophyll: Figure 2a). Here, we use the term "asymmetrical reflectance spectra" to describe spectral asymmetry. Carotenoids strongly absorb blue and green light while allowing some near‐ultraviolet (UVA) light and nearly all long‐wavelength visible light to pass. Many tissues absorb in the UV range, greatly reducing UV reflectance from carotenoid‐based signals (Shawkey et al., 2006; Shawkey & Hill, 2005). Solar illumination also contains less UV overall compared to visible light. Thus, within the visible range, the reflectance of carotenoid‐pigmented signals is much higher at longer wavelengths (Figure 2c,e). In contrast, chlorophyll has two absorption peaks in the blue and red. Therefore, chlorophyll‐pigmented tissue reflects the remaining green light in a relatively symmetrical, Gaussian‐shaped fashion (Figure 2a).

Carotenoid‐based signals change perceived hue with increased pigment concentration because optical absorption varies exponentially with pigment concentration and/or path length (Box 1). Therefore, increasing pigment concentration disproportionately impacts lower reflectance spectral ranges compared to higher‐reflectance ranges (Figure 2b,c). The slope of carotenoid‐colored ornaments' reflectance spectra between ~500 and 700 nm changes substantially as concentration increases. However, as reflectance approaches 0%, these disproportionate relative changes may be perceptually negligible as the material becomes black (Box 1; but see McCoy & Prum, 2019; Davis, Nijhout, & Johnsen, 2020 for discussions of ultra‐low reflectance).

### 3.2 The carotenoid redshift is substantial and perceivable

Carotenoid‐based signals appear redder to birds as concentration increases (Figure 2c–f), and this carotenoid redshift is substantially greater than corresponding changes in a chlorophyll‐pigmented leaf (Figure 2a,b). Passerine birds, a model system for color vision, have at least two kinds of tetrachromacy: UV‐sensitive or not (Vorobyev et al., 1998). Here, we modeled both types of tetrachromacy across four illuminant conditions and found very similar significant results in all cases (see Data S1). We report results for perception in UV‐sensitive tetrachromatic birds under ideal illumination in Figures 1 and 2.

Perceived hue is determined, among many other things, by the photon catches of various color‐sensitive cones in an animal's retina (Maia et al., 2019). In general, leaving aside additional complex phenomena, as the shape of the reflectance curve changes so too does perceived hue. We estimated the quantum catch—the proportion of photons captured by pigments at each photoreceptor—for each color cone in a typical avian UV‐visual system using *vismodel* in the R package *pavo* (Maia et al., 2019), and calculated color distance based on the receptor noise‐limited model (Vorobyev & Osorio, 1998) using the *coldist* function. The simulated carotenoid redshifts produced color distances of 41.2 and 40.9 JNDs (just noticeable differences) as concentration increased from 0.25× to 2×, compared to 7.1 JNDs for chlorophyll over the same concentration range (Figure 2b,d,f). Carotenoid signals led to far greater relative quantum catches by the long‐wavelength sensitive cone as concentration increased compared to chlorophyll (Figure 2b,d,f).

A second metric of perceived hue also showed substantial hue shifts in carotenoids, but little in chlorophyll, as concentration increases (white circles on spectra in Figure 2a,c,e). The wavelength at half‐maximum reflectance is a traditional metric of perceived hue for carotenoids (Montgomerie, 2006), a measure of spectral location in sigmoidal reflectance curves (Andersson et al., 2002; Ospina‐Rozo et al., 2022) that is comparable to the wavelength at maximum reflectance for Gaussian spectra (Maia et al., 2019) such as chlorophyll (Figure 2a,c,e).

In sum, carotenoid‐based signals redshift as concentration increases—changing hue more than found for comparable concentration changes in chlorophyll‐based tissue.

### 3.3 Structures that increase the path length of light also cause a carotenoid redshift

It is important to note that increasing the concentration of the pigment is not the only way to make carotenoids appear redder. In fact, any process that increases light–pigment interactions will have this effect. For example, an animal may increase pigmentary absorption by increasing the path length of light within the pigmented region via scattering by nano‐ or micro‐structural modifications (Section 2.3.2).

#### 3.3.1 In carotenoid‐colored birds, structures drive a perceptually significant carotenoid redshift

In birds, many carotenoid‐pigmented feathers have structural modifications that increase the path length of light through the pigment via scattering (Brush & Seifried, 1968; Hudon, 1991; McCoy et al., 2021; Olson, 1970; Shawkey & Hill, 2005; Surmacki et al., 2016). Brush and Seifried showed that carotenoid‐bearing feathers have flattened, laterally‐compressed barbs lacking barbules (Brush & Seifried, 1968); see also (Hudon, 1991; Olson, 1970). Shawkey and Hill (2005) show that yellow feathers in American goldfinches (*Carduelis tristis*) rely on white structural tissue to appear yellower. When the authors removed structural scattering by index‐matching, the bright yellow feathers appeared “nearly transparent with a faint yellow cast”.

To determine whether structurally‐driven carotenoid redshifts may be perceptually and evolutionarily significant to passerine birds, we analyzed plumage color in the *Ramphocelus* tanagers. These brightly‐colored neotropical birds are a popular model for studies of sexual selection, and both males and females both have red, orange, and yellow carotenoid‐based plumage. Within each species, males and females have approximately the same types and concentrations of the various carotenoid pigments—but males have significantly more saturated colors than females (McCoy et al., 2021). Males achieve these more saturated colors through microstructures that enhance light–pigment interactions in their feathers.

**FIGURE 3:**
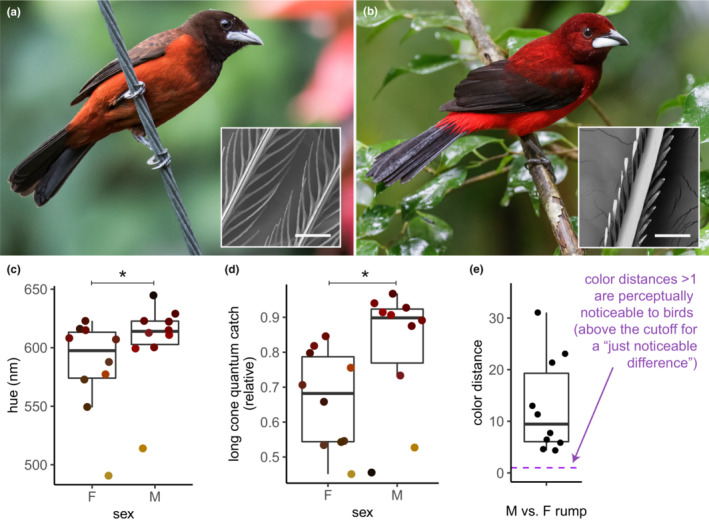
Carotenoid‐based signals redshift, and look redder to birds, when microstructures enhance light–pigment interactions. Here we show a (a) female and (b) male crimson‐backed tanager, *Ramphocelus dimidiatus*, with the associated feather microstructures (insets; scale bar = 100 μm in both). The female feathers have simple microstructures (inset, a) while the males have complex microstructures (inset, b), which focuses more light onto the carotenoid‐colored feather. (c–e) Males have significantly redder plumage than females, even though males and females have significantly correlated carotenoid pigment profiles across *Ramphocelus* tanagers. (c) Male plumage is redder than female plumage, measured as the wavelength at half‐maximum reflectance. Phylogenetic paired *t*‐test: *p*‐value = .0089, phylogenetic mean difference in half‐max‐wavelength = 22.4 nm, 95% CI = (10.2, 34.6). (d). Male plumage appears redder to birds with UV‐sensitive tetrachromatic vision, as modeled by the quantum catch of the long‐wavelength‐sensitive cones under an ideal illuminant (Maia et al., 2019). Phylogenetic paired *t*‐test: *p*‐value = .049; phylogenetic mean difference in long cone quantum catch = 0.15; 95% CI = (0.03, 0.27). Results were significant across four simulated illumination conditions (e.g., shaded forest vs. blue sky) and for non‐UV sensitive birds as well (see Data S1). (e) Compared to female plumage, male plumage is not only redder but also noticeably redder to a UV‐sensitive tetrachromatic bird. All color distances between male and female plumages shown here are perceptually significant to a tetrachromatic bird. See McCoy et al. (2021) for full details of the physical basis of these plumage colors. Spectra analyzed in (c–e) are from Table S2. Photos: Nick Athanas, antpitta.com.

However, the same microstructures that give males more saturated colors should also make males redder. Indeed, males certainly *look* redder than females to human eyes (Figure 3a,b). Color measurements bear out the evidence of our eyes. Males have rumps with reflectance spectra that are significantly redshifted compared to females of the same species (Figure 3c), and visual models show that tetrachromatic birds also perceive male rumps as significantly redder than female rumps (Figure 3d,e). We modeled avian perception by estimating long‐wavelength cone quantum catch for UV‐sensitive tetrachromatic birds and calculating receptor noise–limited (RNL) color distance, under ideal illumination without Von Kries color constancy. Results were consistent when we varied the illuminant (e.g., shaded forest or natural daylight) and avian visual system (UV‐sensitive or not); see Data S1.

This observation—redder males despite the same population of carotenoids—demonstrates that microstructures can enhance light–pigment interactions and therefore produce an observably redder color in carotenoid‐colored birds.

#### 3.3.2 Microstructures and nanostructures increase pigmentary absorption in many living creatures

Beyond carotenoid‐colored birds, many organisms use structures to enhance pigmentary light absorption. Flowers use conical epidermal cells to focus light into the pigmented region, therefore making more saturated petal colors (Gkikas et al., 2015; Gorton & Vogelmann, 1996; Kay et al., 1981; Wilts et al., 2018). For common snapdragons *Antirrhinum majus*, the microstructure of conical epidermal cells—rather than pigment deposition—explains 50%–75% of the variation in absorption between two genetic lines (Gorton & Vogelmann, 1996). Shade‐dwelling rainforest plants appear dark and velvety in color due to conical cells that focus light onto photosynthetic structures within the leaf (Bone et al., 1985). Microstructures focus light onto black pigment to create “super black” or “ultra black” color in peacock spiders (McCoy et al., 2019), stick insects (Maurer et al., 2017), birds (McCoy et al., 2018; McCoy & Prum, 2019), and snakes (Spinner et al., 2013). Ultra‐black fish (Davis, Thomas, et al., 2020) and butterflies (Davis, Nijhout, & Johnsen, 2020; Vukusic et al., 2004; Zhao et al., 2011) use nanostructures to increase scattering and, therefore, light absorption by melanin.

**FIGURE 4:**
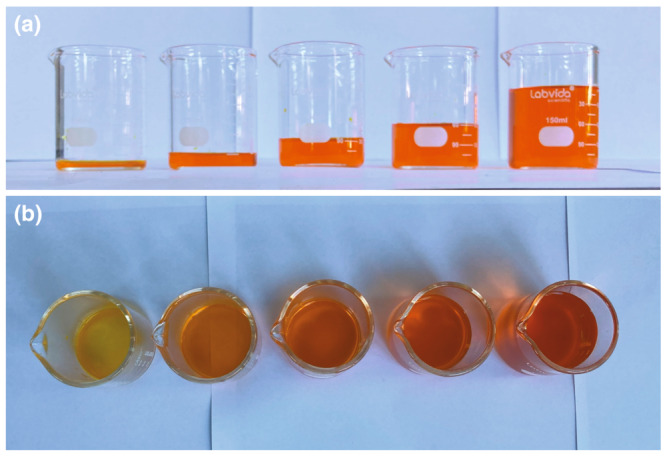
Yellow‐dyed water appears redder when viewed from an angle that results in a longer path length. (a) We dyed water with yellow food coloring in a large beaker, and then we poured the dyed water into five small beakers with different amounts per beaker. Viewed from the side, the beakers all look equally orange because the path length is equal. (b) Viewed from the top, the beakers range from yellow to red‐orange as path length increases from left (shortest) to right (longest).

Path length itself, how far the light must travel through a pigmented region, matters. A yellow‐pigmented solution in a glass beaker appears redder at certain angles due to the differences in path length of light through the solution (Figure 4). The longer the path, the darker, redder, and more saturated the color. Beakers of liquid therefore range from yellow to orange‐red depending on path length (Figure 4). An organism could also increase light–pigment interactions by making a color patch thicker or reflecting light back through the color patch. In this manner, deep‐sea fish have improved photon capture by using elongated rods and reflective tapeta in their eyes (Munk, 1966; Wagner et al., 1998).

### 3.4 Implications for color perception, sexual selection, and honest signaling

For the reasons stated above, a viewer's perception of hue, saturation, and brightness in signals mediated by carotenoids and many other pigments are intimately linked and should be considered together in analyses. The especially asymmetrical reflectance spectra of carotenoid‐based signals have important implications for (i) color perception and (ii) honest signaling in sexual selection.

#### 3.4.1 Color perception and signal quality

It is interesting to speculate that this very property of carotenoid‐based signals—large perceived hue changes alongside brightness and saturation—may make them a more discriminable, and therefore more useful, signal compared to pigments that do not share this property. Hue may be particularly valuable as a signal discriminator. Humans, for example, are better at discerning small hue changes than small saturation changes, although the degree to which hue is a better discriminator depends on the color (Danilova & Mollon, 2016).

Further, once carotenoid‐based signals are established (e.g., to indicate potential mate quality or fruit ripeness and nutritional content; Itle & Kabelka, 2009; Saini et al., 2017), evolution should favor adaptations for sensitive hue‐based discrimination in the range of colors produced by carotenoids: yellows, oranges, and reds. For example, mate‐seekers and fruit‐eaters with this adaptation could therefore more accurately distinguish between high‐ and low‐quality opportunities.

Indeed, many trichromatic and tetrachromatic animals are unusually good at discriminating in the yellow‐orange‐red range—compared to other wavelength ranges—due in part to the placement of the medium and long wavelength‐sensitive cones (Peiponen, 1992; Stockman & Sharpe, 2000). Birds vary in their wavelength discrimination abilities, but it is common to observe peak sensitivities in the orange‐red (560 nm for green‐backed firecrown, *Sephanoides sephaniodes* (Herrera et al., 2008); 555 and 585 nm for black‐chinned hummingbirds, *Archilochus alexandri* (Goldsmith et al., 1981); 460, 540, and 600 nm for pigeon, *Columba livia* (Emmerton & Delhis, 1980); 416, 489 and 557 nm for budgerigar, *Melopsittacus undulates* (Goldsmith & Butler, 2003). Spectral sensitivities often correlate with a species' own plumage colors. Male yellow wagtails (*Motacilla flava*) are most sensitive to yellows, oranges, and reds, while male bluethroats (*Luscinia svecica*) best discriminate orange, purple blue, and purple (Peiponen, 1992).

Behavioral evidence confirms that certain birds with carotenoid‐based signals have sensory adaptations that enhance color discrimination in the red‐orange range. Male zebra finches (*Taeniopygia guttata*) display carotenoid‐pigmented beaks that vary from orange to red and have been interpreted as honest signals (Simons, Briga, et al., 2012). Female zebra finches perceive orange‐red colors in a categorical fashion, but a close relative without carotenoid‐based signals—the Bengalese finches, *Lonchura striata domestica*—do not (Caves et al., 2018, 2021). Interestingly, the zebra finches do not perceive blue‐green colors categorically. Here, categorical color perception may help female zebra finches assess the quality of potential mates' carotenoid‐colored bills, a skill not necessary for the black‐and‐white Bengalese finch. Similarly, many birds, fish, and reptiles have oil droplets that sharpen the absorption spectra of their cones; these droplets are typically yellow, orange, or red and further enhance spectral discrimination in that range (Vorobyev, 2003).

In summary, perhaps carotenoid‐pigmented tissues are particularly well‐suited to serve a signaling function because they have asymmetrical spectra, therefore adding hue as a major axis of signal discrimination alongside brightness and saturation. In response, natural selection may have favored adaptations that enable passerine birds, and perhaps other animals, to sensitively discriminate hues in the red‐orange‐yellow range.

#### 3.4.2 Honest signaling and mechanisms to appear redder

The hue of carotenoid‐colored ornaments also matters for fundamental conclusions about honest signaling theory. In birds, a classic clade for studies of honest signaling and carotenoids, the hue of carotenoid‐colored ornaments is considered an honest signal. Researchers have experimentally observed correlations between redder hued ornaments and higher mate quality as determined by fitness, health, parasite load, or other metrics (Hill et al., 2019; McGraw & Ardia, 2003; Pérez‐Rodríguez et al., 2010; Pike et al., 2007; Weaver et al., 2018). Birds typically ingest carotenoids in their yellow/orange form and then metabolize them to become redder. Therefore, red ornaments have been considered an honest index of proper metabolic function (Weaver et al., 2018). The redness of a male's ornament has been proposed to indicate how well its mitochondria function relative to other males (Hill et al., 2019).

In other words, the redness of a carotenoid‐based signal is an important metric for studies of honest signaling. But carotenoid‐based signals become redder not only through "honest" means, an: any process that increases light–pigment interactions, honest, dishonest, or arbitrary, makes carotenoid‐based ornaments redder in hue. For example, orange‐colored birds that ingest and sequester more orange pigment within the same volume of feathers end up with redder‐hued feathers. Likewise, birds with microstructures that increase the path length of light through carotenoids will also appear redder (Figure 3, Section 2.3).

Above, we analyzed the carotenoid‐based colors in male and female *Ramphocelus* tanagers to show that males are perceivably redder due to feather structures that increase the path length of light.

This alternative pathway to a redder hue—microstructural enhancements—does not immediately fit into models of honest signaling theory, which expect redder colors to correlate with metabolic activity and/or pigment acquisition. There is not enough data on the development and maintenance of microstructures to know whether they are honest, dishonest, or arbitrary. In one study on carotenoids and microstructures, however, microstructures seem not to correlate with condition and quality (Shawkey et al., 2006). Early observations of microstructural modifications in a variety of red‐hued species suggest that they may be common (Brush & Seifried, 1968), and should be investigated further. However, some research has focused on nanostructures (color arising from structures on the size scale of tens or hundreds of nanometers, a smaller size scale than micrometer‐scale microstructures). They found that visual characteristics of nano‐structure‐mediated colors correlate with some measures of quality, potentially because color may depend on developmental regularity (White, 2020).

If females select males based on the proxy of perceived hue, where redness indicates health, perhaps some less‐fit males boost their performance by using microstructures to appear redder without needing to ingest and metabolize more carotenoids. It is difficult to test the “honesty” of such signals. Structural adaptations do not rule out a role for honest signaling models. However, evolutionary theory predicts that if females are judging males based on proxies, males will find ways to cheat the test (McCoy & Haig, 2020). Any proxies invite cheating (Braganza, 2022). For example, guppies cannot synthesize carotenoids but are judged by potential mates based on their carotenoid‐pigmented spots. To make their spots appear redder and more saturated, the males synthesize red pteridine pigments similar in hue to carotenoids (Grether et al., 2001). Male cedar waxwings (*Bombycilla cedrorum*) disproportionately allocate yellow carotenoid pigments to their tail feathers that are most visible to observing females: the central rather than outermost feathers (Surmacki et al., 2016). The more visible feathers are oranger in hue and more saturated in color than the less‐visible feathers. Guppies and cedar waxwings seem to have evolutionary adaptations to cheat in mate choice.

Might structural enhancements that redshift carotenoid ornaments be another male strategy to perform better vis‐à‐vis female choice? More work is required, but by considering the unusual nature of carotenoid‐based signals—which appear redder with more light–pigment interaction—we may be able to gain new insights into the honest, deceptive, and arbitrary signals of sexual selection.

## 4 CONCLUSION AND FUTURE DIRECTIONS

Studies of color perception, honest signaling, and carotenoid‐based ornaments should consider the consequences of an asymmetrical reflectance spectra within the UV–visible range. Hue changes substantially alongside changes in brightness and saturation, so with more light–pigment interactions, carotenoid‐based colors become redder, darker, and more saturated. Carotenoids are not unique in this, but the magnitude of the redshift is greater than corresponding hue changes in, for example, chlorophyll‐pigmented tissue (Figure 2). In any natural system, hue, saturation, and brightness can seldom be changed independently of each other: all three should be considered together.

Further work is needed on the evolutionary trajectories of carotenoid pigment hues. Yellow‐to‐red transitions are well‐documented in bird evolution (Friedman et al., 2011; Ligon et al., 2016), but red‐to‐yellow transitions are rare (McCoy et al., 2021). In these model systems of carotenoid‐colored birds, how common are feather microstructures that increase the path length of light and therefore redshift the color? Carotenoid‐colored feathers evolved many times independently in birds (Thomas et al., 2014); do such microstructures evolve before, after, or concurrently with carotenoid signals? Such questions will help determine whether carotenoid signals honestly indicate quality or are gamed by lower quality males with structural amplifiers.

In closing, it is interesting to note, however, that some carotenoids in animals appear blue, purple, or pink due to the orientation of carotenoid molecules and the interaction of carotenoids with associated proteins (Shawkey & D'Alba, 2017). European lobsters (*Homarus gammarus*), for example, appear blue in the wild due to a caroteprotein called α‐crustacyanin that binds carotenoid pigments and blue‐shifts the color. Boiling causes the protein to denature, and the free carotenoids therefore appear red (Britton & Goodwin, 1982). In birds, the head‐to‐tail alignment of canthaxanthin carotenoid molecules in birds feathers influences whether the resulting color appears red (as in the scarlet ibis, *Eudocimus ruber*, Threskiornithidae), orange‐red (summer tanager, *Piranga rubra*, Cardinalidae), or violet‐purple (white‐browed purpletuft, *Iodopleura isabellae*, Tityridae) (Mendes‐Pinto et al., 2012). These and other chemical effects dramatically change hue but receive comparatively little attention in evolutionary research.

## AUTHOR CONTRIBUTIONS

**Dakota E. McCoy:** Conceptualization (lead); data curation (lead); formal analysis (equal); funding acquisition (equal); investigation (equal); methodology (equal); resources (equal); software (equal); validation (equal); visualization (equal); writing – original draft (equal); writing – review and editing (equal). **Allison J. Shultz:** Conceptualization (equal); data curation (equal); formal analysis (equal); investigation (equal); resources (equal); supervision (equal); writing – review and editing (equal). **Jacqueline E. Dall:** Conceptualization (supporting); data curation (supporting); methodology (supporting); resources (equal); writing – review and editing (supporting). **Jennifer A. Dionne:** Conceptualization (equal); formal analysis (equal); funding acquisition (equal); investigation (equal); methodology (equal); project administration (equal); resources (equal); supervision (equal); validation (equal); visualization (equal); writing – original draft (equal); writing – review and editing (equal). **Sönke Johnsen:** Conceptualization (equal); data curation (equal); formal analysis (equal); funding acquisition (equal); investigation (equal); methodology (equal); project administration (lead); resources (equal); software (equal); supervision (equal); validation (equal); visualization (equal); writing – original draft (equal); writing – review and editing (equal).

## FUNDING INFORMATION

Stanford Science Fellowship (DEM). NSF Postdoctoral Research Fellowships in Biology PRFB Program, grant 2109465 (DEM).

## Supporting information


Table S1

Table S2

Appendix S1

Data S1

Data S2

## Data Availability

All data and code are available in the main text or the supplementary materials: Tables S1 and S2, Data S1 and S2.
