# Supplementary material for: The carotenoid redshift: Physical basis and implications for visual signaling
Source: Ecol Evol. 2023 Sep 7;13(9):e10408. doi: 10.1002/ece3.10408 (PMC10485323; doi:10.1002/ece3.10408)
Supplement: Supplementary file 3 — Appendix S1 [file ECE3-13-e10408-s002.docx]

Supplementary Table 1: Reflectance measurements for tanager birds (*Ramphocelus* spp. males and females) from McCoy et al. 2021, analyzed in Figures 2-3.

Supplementary Table 2: Reflectance measurement for a green leaf from Johnsen *et al.*, 2006m analyzed in Figure 2.
